# On the Effects of Food on the Blood

**Published:** 1846-03

**Authors:** 


*On the Effects of Food on the Blood.*—Dr. Buchanan has drawn the following conclusions on the state of the blood after taking food:

1. The serum of the blood of a healthy man fasting, is perfectly transparent, and of a yellowish or slightly greenish tint.

2. A heterogeneous meal, such as that usually set on the tables of the rich, renders the serum white.

3. The whiteness may commence as early as half an hour after eating, and may continue ten or twelve, and sometimes as long as eighteen hours, according to the kind and quality of the food, and the state of the functions of the primary and secondary digestion.

4. *Starch*, or *sugar*, probably all vegetable substances destitute of oil, give no whiteness to the serum of the blood.

5. *Fibrin*, *albumen*, and *casein*, and probably *protein compounds* in all their forms, if destitute of oil, give no whiteness.

6. Oils combined, whether naturally or artificially, with protein compounds or with starch, render the serum of the blood white; probably, therefore, oils produce that effect in whatever way taken.

7. Gelatin seems to render the serum of the blood white; this, however, cannot be considered as certainly established, as there may have been some fat in the beef tea which was taken along with the calf-foot jelly in both experiments on which the above conclusion rests.

8. That coagulum of the blood very frequently exhibits, after taking food, a crust of pellucid fibrin, or of pellucid fibrin dotted with more opaque particles, and with little of the contraction technically named "cupping."

9. The appearances of the coagulum just mentioned are much more common after azotized than after non-azotized food.

These conclusions relating to the visible characters of the blood may be considered, with the single exception above mentioned, as well established. The conclusions which follow relate chiefly to the chemical properties of the blood, and are not worthy of the same reliance; but the evidence on which they rest has been laid before the reader, and he must judge of them for himself

1. The substance defined above under the name of Pabulin, is most abundant in the blood a few hours after taking food, sooner or later according to the rapidity of digestion.

2. It is less abundant as the time when the food has been taken is more remote, and is small in quantity after a fast of twenty-four hours.

3. It is much more abundant after azotized, than after non-azotized food.

4. It varies in quality, floating or subsiding, according to the kind of food taken.

5. It is probably analogous in nature to the white substance which gives color to the serum of the blood.

6. The difference between these two forms of this substance probably is, that it is sometimes combined with an alkaline, or earthy salt (chloride of sodium, sulphate of soda, &c.,) and sometimes with an oily body (stearate of glycerine, &c.) In the former case it seems to dissolve completely in the blood, while in the latter it is only partially dissolved, and renders the serum opaque.

7. The azotized principles of the food are probably made to combine, in the digestive tube, with the alkaline, earthy, and oily salts mentioned above; and thus become capable of being absorbed into the blood.

8. The alkaline and earthy compounds are probably absorbed directly by the blood-vessels, while it seems to be well ascertained that the oily compounds are absorbed through the lacteals.—*London Medical Gazette.*

